# Cone Beam Computed Tomographic Analysis of the Shape, Height, and Location of the Mandibular Lingula in a Population of Children

**DOI:** 10.1155/2013/825453

**Published:** 2013-11-20

**Authors:** Ahmet Ercan Sekerci, Kenan Cantekin, Mustafa Aydinbelge

**Affiliations:** ^1^Department of Oral Department of Oral and Maxillofacial Radiology, Faculty of Dentistry, Erciyes University, 38039 Kayseri, Turkey; ^2^Department of Pedodontics, Faculty of Dentistry, Erciyes University, 38039 Kayseri, Turkey

## Abstract

*Objectives*. This is the first study to identify and classify the different morphological shapes of the mandibular lingula (ML) in children using cone-beam computed tomography (CBCT). *Material and Methods*. A retrospective study was performed to evaluate the shape, height, and location of the ML in relation to the surrounding structures using CBCT images of mandibles obtained from 269 children. The shape of the ML was classified into triangular, truncated, nodular, or assimilated types. The location was determined by five distances. The height of the lingula was also measured from the lingular tip to the mandibular foramen. *Results*. A nodular shape of the ML was most commonly found (48.3%, *n* = 260) followed by truncated (23.4%, *n* = 126), assimilated (14.4%, *n* = 78), and triangular (13.7%, *n* = 74). The mean distance of ML from the anterior and posterior borders of mandibular ramus was 13.3 ± 2.3 mm and 10.2 ± 1.6 mm, respectively. In the majority of the mandibles studied, the ML was located above the occlusal plane. *Conclusion*. The present study provides new information to the literature concerning the shape, height, and location of the lingula in a Turkish pediatric population. This finding may assist clinicians to localize the lingula and avoid intraoperative complications.

## 1. Introduction

The objectives of the present investigation are to determine the shape, height, and location of the lingula in relation to the mandibular ramal landmarks and the mandibular first molar in a Turkish pediatric population of 6- to 12-year-old children using cone-beam computed tomography (CBCT). The differences in the parameters were also evaluated between the sexes. Comparisons with previous studies of various ethnic and racial groups were also assessed.

The lingula is a tongue-shaped bony projection on the medical surface of the mandibular ramus close to the posterior margin of the mandibular foramen [1]. The exact location of the mandibular foramen on radiographs is not always easy to be established due to its radiolucency and the superimposition of contralateral mandibular structures [2]. The mandibular foramen has often considered to be the most reliable reference point for approaching the inferior alveolar nerve in several anesthesia techniques, which led to several studies about its position and its anatomical relationships to clinically recognizable landmarks. It has been speculated that the mandibular lingula and foramen change the ratio of their positions on the ramus of growing children [3]. Tsai [4] observed in children a variation in the difference between the distance from the mandibular foramen to the anterior border and to the posterior border. This variation is caused by regional growth in different directions in each of Hellman's dental developmental stages [3].

Because of its connection to nerve and vascular structures the study of the lingula features provides significant information related to oral and maxillofacial surgical procedures, such as the sagittal split ramus osteotomy, vertical ramus osteotomy, inverted L osteotomy [2], orthognathic surgery, mandibular trauma management, eradication of benign and malignant lesions, preprosthetic surgery, and nerve injury during inferior mandibular nerve block [3]. The lingula is used for identifying the site for injection of local anaesthetics or for excision of nerve for facial neuralgia [4]. If oral-maxillofacial surgeons are unable to identify the lingula correctly, intraoperative complications such as hemorrhage, unfavorable fracture nerve injury and may occur [5, 6].

The present study is unique in that the mandibles were collected from a single ethnic group (Turkish) with known ages and gender. The number of samples in the present study was large enough to compare the shape of the lingula and its distribution in addition to determining the location of the lingula. The subjects (both girls and boys) have a known and similar age range and the results were subjected to statistical analyses. It was therefore possible to investigate sex and side differences.

The researchers analyzed the morphological characteristics of the lingula, and they stated at the conclusion that such structural variability could account for failure to block the inferior alveolar nerve [7, 8]. Variations in the shape of the lingula have been reported by various authors [9–11]; for example, Tuli et al. [5, 6] classified lingula into four different types based on its shape, namely, triangular, truncated, nodular, and assimilated types.

## 2. Material and Methods

The study protocol was approved by the Ethics Board of the Medical Faculty of Erciyes University. We designed a retrospective study composed of CBCT images of 2,103 patients who are presented to the Dentomaxillofacial Radiology service at the Erciyes University, Dentistry Faculty. 

**Figure 1:**
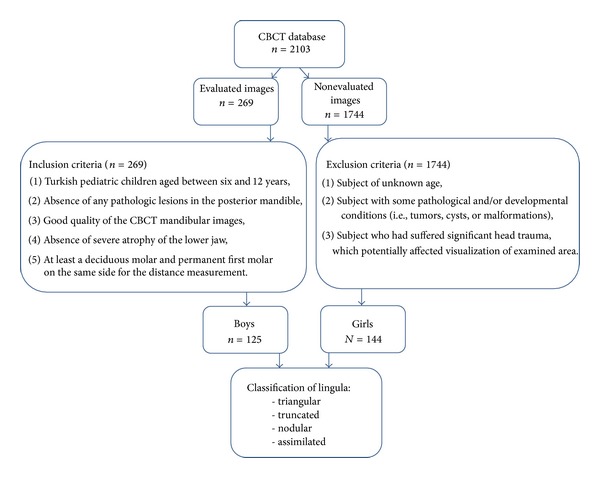
Schematic illustration of the preparation of the samples was demonstrated.

A schematic illustration of the preparation of the samples is demonstrated in Figure 1. All 269 patients had been referred for CBCT diagnosis and treatment planning and consisted of 19 impacted tooth patients, 178 orthodontic patients, 25 possible pathosis patients, 27 supernumerary tooth patients, and 20 temporomandibular joint disorder patients (Table 1).

**Figure 2:**
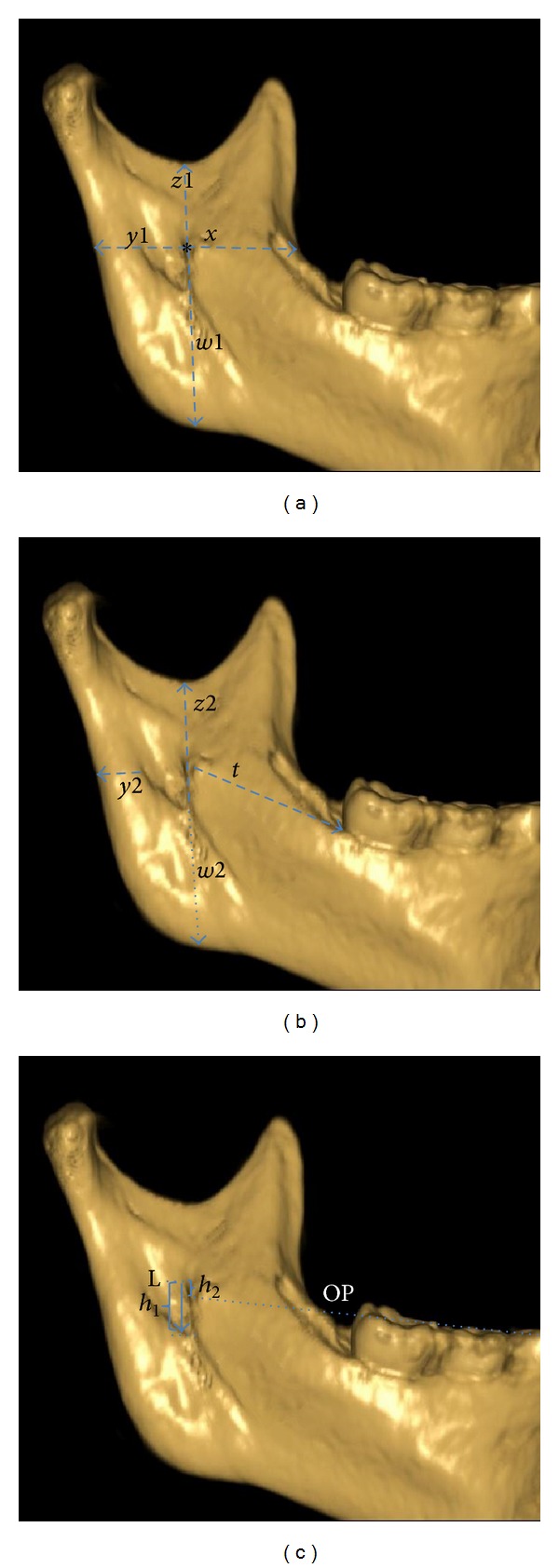
Three-dimensional CBCT image of the internal surface of the mandible showing the measurement for localizing the position of the lingula, entrance of mandibular foramen from the various landmarks. The “*x*” measurement indicated the distance in millimetres found between the most anterior part of the mandibular lingula points to the anterior border of the mandibular ramus in a straight horizontal line; the “*y*” measurement represented the distance between the most posterior part of the mandibular lingula and foramen points to the posterior border of the mandibular ramus in the same horizontal orientation; the “*w*” measurement determined the distance between the lower point of the mandibular foramen and lingula points to the mandibular base in a vertical straight line; the “*t*” measurement determined the distance most anterior part of the mandibular lingula points to the alveolar socket of first mandibular molar tooth; the “*z*” measurement determined the distance between the same initial points as for the *w* distance to the lower point of the sigmoid notch. The horizontal distances were measured parallel to the occlusal plane (OP) of the molars, whereas the vertical distances were measured perpendicular to the occlusal plane of the molars. The ratio of (*x*) to (*x* + *y*1) was also calculated and used as an additional guide to localize the lingula [21]. Vertical distance from the tip of the lingula to the lower border of the mandibular foramen was measured as height of the lingula (*h*_1_). The measurement included the distance from the lingula to the occlusal plane of the molars (*h*_2_).

The shapes of the lingula were classified using the classification proposed by Tsai [4]. To determine the exact location of lingula, the distance of the tip of the lingula from various mandibular ramal landmarks was measured (Figures 2(a)–2(c)). 

The CBCT mandibular images were analyzed in the NNT viewer which is a simple version of the NNT software of the CBCT (Newtom 5G, QR, Verona, Italy) machine in a Dell Precision T5400 workstation (Dell, Round Rock, TX, USA) and a 32-inch Dell LCD screen with a resolution of 1280 × 1024 pixels in a darkroom. The contrast and brightness of the images were adjusted using the image processing tool in the software to ensure optimal visualization. All the measurements were carried out by the same person (AES).

*Statistical Analyses.* The values obtained were tabulated; and the mean average and respective standard deviations (SDs) were calculated for all distances studied. The data analyses were performed by using the Statistical Package for the Social Sciences (SPSS), version 16.0, (SPSS Inc., Chicago, Ill) and statistical significance was determined at the level of *P* < .05. The distances were calculated for each of the measurements on right and left sides, and a comparison of the mean values of the right and left sides were made by using *t*-tests. 

## 3. Results

The study subjects consisted of 144 (53.5%) girls and 125 (46.5%) boys. The mean age of the patients was 9.17 (SD: 1.87), with ages ranging from 6 to 12 years.

**Figure 3:**
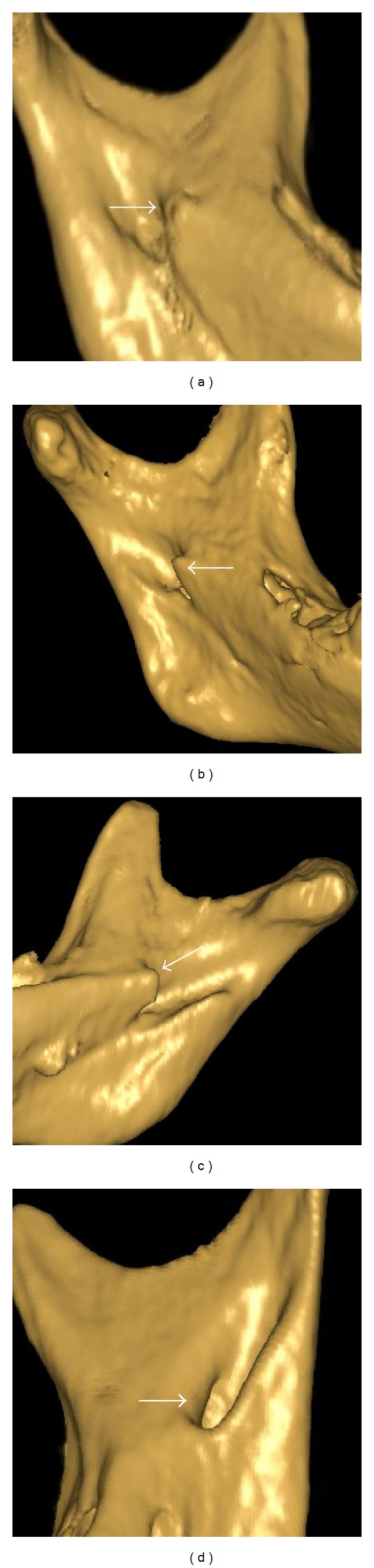
Different shapes of lingulae (a) nodular; (b) triangular; (c) truncated; (d) assimilated.

The most common shape was the nodular shape of the ML (48.3%, *n* = 260), followed by truncated (23.4%, *n* = 126), assimilated (14.4%, *n* = 78), and triangular (13.7%, *n* = 74) (Figures 3(a)–3(d)). A bilateral shape (65.7%) was found more often than a unilateral one (34.3%). The MLs were found bilaterally as nodular in 92 sides (52%), truncated in 41 sides (23.2%), triangular in 23 sides (13.0%), and assimilated in 21 sides (1.9%). The distribution of the lingular shapes was also compared between girls and boys (Table 2); there was a statistical difference between gender in triangular and nodular shape.

The height, distance of ML from various mandibular ramal landmarks as well as from the distal side of the alveolar socket of the mandibular permanent first molar tooth and ML ratio are shown in Table 3. The ML was located at 13.3 ± 2.3 mm from the anterior border of mandibular ramus, 10.2 ± 1.6 mm from the posterior border of the ramus, and 11.4 ± 2.5 mm from the mandibular notch. The mean distance of the ML from the distal surface of the alveolar socket of the mandibular permanent first molar tooth was 24.7 ±3.7 mm. The mean height (*h*_1_) of the ML was 5.3 ± 1.6 mm, and the mean ML ratio was determined out to be 0.55 ± 0.04. Statistically significant difference was shown in Table 3. The lingulae were 14.6% (79) at the same level with the occlusal plane, 57.9% (312) above the plane, and 27.5% (97) below the plane.

## 4. Discussion

This is the first study to systematically evaluate the different morphological shapes of the ML in a pediatric population using CBCT images. 

Pain control during dental procedures is very important in children to maintain a positive relationship between the child and dentist building trust and allaying fear and anxiety [12]. The inferior alveolar nerve block is the most common technique for providing local anesthesia [13] before restorative and surgical procedures of the mandibular posterior teeth [14]. This technique provides anesthesia of teeth, jaw, lip, gingiva, and mucous membrane up to the midline at the related part. However, Malamed identifies the inferior alveolar nerve block as the injection with the highest clinical failure rate, which he reports to be 15 to 20 percent when properly administered [15]. This high failure rate is often attributed to a high degree of variation in the morphology of the mandibular ramus and the location of the ML, especially in childhood and adolescence [16]. Therefore, defining the anatomical characteristics of the ML region plays an important role in successful anesthesia for dental and surgical procedures.

Several factors contribute to the reliability of landmark identification in children: the density and sharpness of the images, the anatomic complexity and superimposition of hard and soft tissues, the definition of the landmark, and the training level or experience of the observers [17, 18]. CBCT in dentistry has provided an imaging solution that has neither the projection errors associated with magnification nor the superimposition problems associated with traditional panoramic imaging [19]. In addition, CBCT has a wide range of tools, such as 3D reconstructions in any direction to permit the accurate identification of landmarks. Studies have reported excellent accuracy with 3D computed tomography (CT) [20]. Using CBCT (3D) in our study, identification of the AMF reflected a real clinical situation.

The frequency of different morphological types of lingula studied by different authors varied among populations and races [1, 5, 6, 21–26] (Table 4). Triangular and truncated shapes of the ML were found most commonly in previous studies [4]. In the present study, a total of 261 nodular shapes were detected in 168 of the 269 patients, and the frequency rate was found to be 48.3%, which differed from the rates generally reported in previous studies.

Tuli et al. [5, 6] observed gender variation of the lingula shapes in their specimens, and in their study, the triangular and assimilated types were the most common and the least common types in males (67.9% and 5%) and females (70.6% and 4.4%), respectively. They observed the truncated type twice as often in males (17.6% sides) as in females (8.8%), and the nodular type was a little less than double in females (16.2%) as compared with males (9.6%). In Jansisyanont et al.'s [21] study, there was a different genderwise variation observed. Of the 74 lingulae of mandibles belonging to females, from 60 lingulae, the triangular type was found in 21.7%, truncated in 23.3%, nodular in 33.3%, and assimilated in 21.7%, of cases. In case of males, the triangular type was observed in 36.5%, truncated in 31.1%, nodular in 27% and assimilated in 5.4% of cases. In our study, there was statistical difference between gender in the triangular and nodular shapes. The nodular and triangular types were the most and least prevalent types in boys and girls.

The height level of the mandibular foramen is an important reference for the inferior alveolar nerve block. Kanno et al. [3] found the mandibular lingula position to be 6 mm above the occlusal plane in 7- to 8-year-old children and 10 mm in 9- to 10- year-old children. Nicholson [7] found the foramen located to be below the occlusal surfaces of the molar teeth. Murlimanju et al. [23] reported in 38 dry, adult, Negroid, Zimbabwean mandibles that 47.1% were at the same level with the occlusal plane, 29.4% were above the and 23.5% were below the plane. Hwang et al. [27] reported that the location of the mandibular foramen changed with age, and that in children, it was located below the occlusal plane, while in adults it was 4.16 mm above the occlusal plane. In Kositbowornchai et al.'s [24] study, the mandibular foramen, measured from three-dimensional CBCT images, was 10 mm above the occlusal plane. In the present study, the mean height level (*h*_2_) was measured to be 2.0 ± 1.2 mm above the occlusal plane. A study on Thai mandibles [28] showed that the lingular heights on the right and left sides were 8.7 ± 2.0 mm and 8.2 ± 2.1 mm, respectively. Another study in the Thai population by Jansisyanont et al. [21] reported the height of the lingula to be 8.2 ± 2.3 mm. In the present study, we defined the distance as 5.3 ±1.6 in the pediatric population. Woo et al. [29] reported a study on a Korean population, in which the height of lingula was found to be higher, that is, 10.51 ± 3.84 mm. In a study reported by Samanta and Kharb [30], the lingular height was found to be 5.5 ± 2.02 mm. Another study in a Brazilian population by Monnazzi et al. [2] reported the height of lingula to be 5.82 ± 0.43 mm. Nicholson [7] studied eighty dry, adult human mandibles of East Indian ethnic origin and reported a height of the lingula on the right side to be 8.6 ±4.7 mm and left side to be 9.1 ± 5.7 mm. In the present study, the mean height of the lingula was 5.3 ± 1.6 mm, with a statistical difference between gender on right and left sides (Table 3).

The location of the lingula varies among the various ethnic and racial groups [7, 24, 31, 32]. Nevertheless, the common trend was that the distances in females were shorter than or nearly equal to those found in males. This observation is consistent with the knowledge that females generally have larger mandibles than males [30]. In the present study, the mean distance of the lingula to the anterior border of mandibular ramus was 13.3 ± 2.3 mm. The distance of the lingula from the other mandibular ramal landmarks observed in the present study varied from those reported in various populations as shown in Table 5. 

Jansisyanont et al. [21] and the study by Afsar et al. [31] reported that there was no difference in relation to the mandibular ramal landmarks and the mandibular second molar when comparing sex and side groups except for the distance from the lingula to the distal surface of the mandibular second molar of the female left and the male right groups. In the present study, the statistical differences are shown in Table 3.

Although the radiation doses from CBCT are significantly lower than in medical CT, they are generally higher than conventional dental radiography [33]. Recently, the SEDENTEXCT working group proposed provisional evidence-based selection criteria with clinical indications regarding when CBCT should be performed. CBCT should only be used when the clinical question cannot be answered by conventional radiography, and the field of view (FOV) should be limited to the region of interest [33]. Ideally, CBCT equipment should be able to offer a choice of volume sizes to reduce patients' radiation exposure levels. A risk-benefit analysis must be performed on each individual patient when CBCT is being considered, and in order to assess the risk of CBCT, the effective dose must first be calculated as well.

## 5. Conclusion

 The present study provides new information to the literature concerning the shape, height, and location of the mandibular lingual in the Turkish pediatric population. The findings of the present study could be utilized in clinical and dental procedures to localize the lingula and avoid intraoperative complications. The bilateral nodular shape of the lingula was most common in the whole population of study and in each sex. The mean height of the lingula was 5.3 mm. The lingula was located an average of 13.3 mm from the anterior border of the mandibular ramus, 11.4 mm from the mandibular notch, and 24.7 mm from the distal surface of the mandibular permanent first molar. The landmarks for the mandibular nerve block are important for efficient anesthesia during dental treatments. The results from the present study suggest that clinicians or oral surgeons should insert a needle approximately 13.3 mm from the anterior border of the ramus, and approximately 2.0 mm above the occlusal plane due to fact that the lingula in the majority of the samples was found above the occlusal plane. 

## Figures and Tables

**Table 1:** Description of the 269 subjects and their indications for cone beam CT (CBCT) referral.

Reason for Scan	No. of Subjects
Impaction localization	19
Orthodontic records	178
Other possible pathosis	25
Supernumerary teeth localization	27
TMJ assessment	20

**Table 2:** Distribution and incidence (in parentheses) of lingula in girls and boys, bilateral or unilateral.

Type	Shape	Girl			Boy				Total				
		(*n* = 144)			(*n* = 125)			*P* value	Bilateral	Unilateral (*n* = 85)		Right	Left
		Bilateral	Unilateral	Total	Bilateral	Unilateral	Total		(*n* = 177)	Right	Left	269	269
1	Triangular	15 (10.4)	20 (6.9)	50	8 (6.4)	8 (3.2)	24	0,021*	23 (13.0)	16 (17.6)	12 (13.2)	39 (14.5)	35 (13.0)
2	Truncated	17 (11.8)	32 (11.1)	66	24 (19.2)	12 (4.8)	60	0,767	41(23.2)	40 (44.0)	4 (4.4)	81 (30.1)	45 (16.7)
3	Nodular	36 (25.0)	52 (18.1)	124	57 (44.8)	23 (9.2)	137	0,011*	93 (52.5)	16 (17.6)	59 (64.8)	108 (40.1)	152 ( 56.5)
4	Assimilated	12 (8.3)	24 (8.3)	48	9 (9.6)	11 (4.4)	29	0,069	21 (11.9)	19 (20.9)	16 (17.6)	41 (15.2)	37 (13.8)

*Statistically significance.

**Table 3:** Distance of lingula from various ramal landmarks of 269 mandibula; distance from distal side of the alveolar socket of mandibular permanent first molar tooth and lingula ratio with their comparison between gender sides.

	Side	Gender	Minimum (mm)	Maximum (mm)	Mean (mm)	Std. deviation	*P* value	Total	
								Mean (mm)	Std. deviation
Distance from anterior border of ramus	*x*-right	1	10,1	19,4	13,6	2,5	0,13	13,3	2,3
		0	9	16,1	12,7	1,9			
	*x*-left	1	10,2	19,5	14,1	2,1	0,054		
		0	9,3	18,9	12,8	2,5			

Distance from posterior border of ramus	*y*1-right	1	7,5	13,2	10,9	1,6	0,000*	10,2	1,6
		0	6,1	12,1	9,2	1,7			
	*y*1-left	1	8,7	13,8	11,3	1,5	0,015*		
		0	7,6	12,8	10,2	1,5			
	*y*2-right	1	4,2	10,7	7,0	2,3	0,019*	6,3	2,1
		0	2,6	9,9	5,6	1,9			
	*y*2-left	1	3,2	11,2	7,2	2,3	0,026*		
		0	2,4	10,6	5,8	1,9			

Distance from mandibular notch	*z*1-right	1	8,2	18,2	12,6	2,5	0,06	11,4	2,5
		0	7	17,6	11,2	2,5			
	*z*1-left	1	7,2	16,2	12,4	2,5	0,014*		
		0	6,3	15,7	10,6	2,7			
	*z*2-right	1	13,2	24,4	17,9	3,0	0,041*	16,7	3,4
		0	8,2	23,6	15,7	4,3			
	*z*2-left	1	8,4	22,5	17,8	3,2	0,005*		
		0	6,6	22,5	15,0	3,7			

Distance from mandibular base	*w*1-right	1	19,9	28,1	23,6	2,2	0,039*	23,1	3,2
		0	11,9	25,9	21,7	4,0			
	*w*1-left	1	14,7	27,8	23,9	3,0	0,56		
		0	11,9	26,3	23,5	3,0			
	*w*2-right	1	12,5	23,8	18,3	2,8	0,29	17,9	3,0
		0	12,2	24,3	17,4	3,2			
	*w*2-left	1	11,9	23,4	18,2	2,9	0,71		
		0	12,7	22,8	17,9	3,0			

Distance from distal side of the alveolar socket of 1st mandibular molar tooth	*t*-right	1	18,1	31,1	24,9	3,8	0,23	24,7	3,7
		0	16,5	29,4	23,7	3,4			
	*t*-left	1	16,1	32	25,5	4,2	0,41		
		0	17,9	30,9	24,7	3,5			

Height of the lingula	*h*1-right	1	3	9,7	5,6	1,4	0,027*	5,3	1,6
		0	2,3	8,3	4,7	1,5			
	*h*1-left	1	3,6	9,6	6,3	1,6	0,000*		
		0	2,7	8,8	4,7	1,5			
	*h*2-right	1	1,3	5,1	2,1	0,5	0,74	2,0	1,2
		0	0,3	5,1	1,9	1,5			
	*h*2-left	1	0,2	4,7	2,2	1,1	0,2		
		0	0,2	4,6	1,6	1,3			

Lingula ratio: *x*/*x* + *y*1 (%)	right	1	0,45	0,59	0,54	0,04	0,48	0,55	0.047
		0	0,51	0,63	0,58	0,05			
	left	1	0,44	0,61	0,53	0,05	0,52		
		0	0,49	0,62	0,55	0,048			

Groups: 1: boy, 0: girl.

*Statistically significance.

**Table 4:** The most prevalent shape of lingula in reported studies.

Authors	Reference	Year	Population
Tuli et al.	[5]	2000	Triangular
Devi et al.	[1]	2003	Truncated
Hossain et al.	[22]	2001	Triangular
Murlimanju et al.	[23]	2012	Triangular
Kositbowornchai et al.	[24]	2007	Truncated
Nirmale et al.	[25]	2012	Triangular
Lopes et al.	[26]	2010	Triangular
Jansisyanont et al.	[21]	2009	Truncated
Present study			Nodular

**Table 5:** Comparison of various studies on location of lingual.

Authors	Reference	Study design	Population	Year	Distance from anterior border of ramus (mm)	Distance from posterior border of ramus (mm)	Distance from mandibular notch (mm)
Woo et al.	[29]	Dry mandible	Korea	2002	18.6 ± 2.5	16.1 ± 3.5	19.8 ± 5.1
Kositbowornchai et al.	[24]		Thai	2007	20.7 ± 2.8	15.4 ± 1.9	—
Jansisyanont et al.	[21]		Thai	2009	20.6 ± 3.5	18.0 ± 2.6	16.6 ± 2.9
Samanta and Kharb	[30]		India	2012	20.0 ± 2.4	15.0 ± 2.7	15.4 ± 2.7
Monnazzi et al.	[2]		Brazil	2012	16.5 ± 2.3	14.6 ± 2.13	16.4 ± 2.6
Present study		CBCT	Turkey	2013	13.3 ± 2.3	10.2 ± 1.6	11.4 ± 2.5
